# Contingency Management interventions for non-prescribed drug use during treatment for opiate addiction: A systematic review and meta-analysis

**DOI:** 10.1016/j.drugalcdep.2017.05.028

**Published:** 2017-09-01

**Authors:** Tom S. Ainscough, Ann McNeill, John Strang, Robert Calder, Leonie S. Brose

**Affiliations:** a Addictions Department, Institute of Psychiatry, Psychology and Neuroscience, King’s College London, London, UK; b UK Centre for Tobacco and Alcohol Studies, UK

**Keywords:** Meta-analysis, Contingency management, Opiates, Cocaine, Tobacco, Polysubstance, Reinforcement

## Abstract

**Background and aims:**

Use of non-prescribed drugs during treatment for opiate addiction reduces treatment success, creating a need for effective interventions. This review aimed to assess the efficacy of contingency management, a behavioural treatment that uses rewards to encourage desired behaviours, for treating non-prescribed drug use during opiate addiction treatment.

**Methods:**

A systematic search of the databases Embase, PsychInfo, PsychArticles and Medline from inception to March 2015 was performed. Random effects meta-analysis tested the use of contingency management to treat the use of drugs during opiate addiction treatment, using either longest duration of abstinence (LDA) or percentage of negative samples (PNS). Random effects moderator analyses were performed for six potential moderators: drug targeted for intervention, decade in which the study was carried out, study quality, intervention duration, type of reinforcer, and form of opiate treatment.

**Results:**

The search returned 3860 papers; 22 studies met inclusion criteria and were meta-analysed. Follow-up data was only available for three studies, so all analyses used end of treatment data. Contingency management performed significantly better than control in reducing drug use measured using LDA (*d* = 0.57, 95% CI: 0.42–0.72) or PNS (*d* = 0.41) (95% CI: 0.28–0.54). This was true for all drugs other than opiates. The only significant moderator was drug targeted (LDA: Q = 10.75, *p *= 0.03).

**Conclusion:**

Contingency management appears to be efficacious for treating most drug use during treatment for opiate addiction. Further research is required to ascertain the full effects of moderating variables, and longer term effects.

## Highlights

• Contingency management (CM) reduces other drug use in opiate addiction treatment.
• Meta-analyses did not find evidence of effectiveness for non-prescribed opiate use.
• CM is effective for cocaine, tobacco, opiates + cocaine, tobacco, polysubstance use.
• Evidence is lacking for long-term effects.

## 1 Introduction

Amongst those in treatment for opiate addiction, use of non-prescribed drugs is very common. Hair samples from 99 recently deceased opiate addiction patients identified a range of 21 different drugs being used during treatment, including cocaine, amphetamine, morphine and diazepam (Nielsen et al., 2015). Other studies have observed that over a third of patients entering opiate addiction treatment were also DSM-IV dependent on a drug other than heroin (not including nicotine) (Puigdollers et al., 2009), and poly drug use has been reported to be as high as 68% (Taylor, 2015). These high levels of drug use are not limited to illicit substances. Tobacco smoking is highly prevalent in drug treatment in general (Cookson et al., 2014), with prevalence rates of over 90% observed in individuals undergoing methadone treatment for opiate addiction (Best et al., 2009, Clemmey et al., 1997). Methadone itself has been linked to increased tobacco cigarette consumption, smoke intake and self-reported satisfaction of cigarette smoking (Chait and Griffiths, 1984), and to increased alcohol consumption compared with heroin use (Backmund et al., 2003).

Use of non-prescribed drugs during methadone treatment for opiate addiction has been associated with a range of adverse effects such as poor treatment retention and outcomes (Magura et al., 1998). Use of a single drug during opiate addiction treatment is associated with a threefold greater risk of dropping out of treatment, and use of multiple drugs quadruples the risk of dropping out (White et al., 2014). For example, cocaine use during methadone treatment has been linked to persistence of heroin use (Hartel et al., 2011). Similarly, tobacco smoking during opiate detoxification results in significantly greater opiate craving and significantly lower rates of detoxification completion (Mannelli et al., 2013) and is associated with higher levels of illicit drug use (Frosch et al., 2000).

High prevalence rates and the links to adverse treatment outcomes indicate a need for effective interventions for non-prescribed drug use during opiate addiction treatment. One of the most widely used behavioural interventions is contingency management (CM). CM is based on the theory of operant conditioning (Skinner, 1938), which states that the administering of a reward for a particular behaviour increases the likelihood of that behaviour being repeated. In the current context, CM uses rewards (vouchers, clinical privileges or desirable items to be won as prizes for example) to positively reinforce abstinence from or reduced use of drugs during treatment for opiate addiction. CM differs from other common psychological interventions in that the focus of treatment is not on introspective analysis of discrepancies between goals and behaviour (as in motivational interviewing) or modification of flawed cognitive processing (as in CBT), but instead on directly influencing the reinforcement mechanisms involved in addiction (Jhanjee, 2014). Previous reviews have shown CM to be moderately effective in treating substance use (illicit drugs, alcohol and tobacco) disorders in general (Benishek et al., 2014, Davis et al., 2016, Dutra et al., 2008, Lussier et al., 2006, Prendergast et al., 2006), particularly so for opiate addiction (Prendergast et al., 2006). Despite a number of recent reviews assessing the efficacy of CM for substance use in general, very little is known about the use of CM for treating use of non-prescribed drugs in the context of opiate addiction treatment, where treatment outcomes may differ.

Whilst some of these reviews included studies assessing the use of CM in this context (Benishek et al., 2014, Castells et al., 2009, Davis et al., 2016, Lussier et al., 2006), none directly addressed the efficacy of CM for substance use during opiate addiction treatment. The most recent review of this specific use of CM is a meta-analysis published over 16 years ago (Griffith et al., 2000). CM was observed to perform better overall than control, and the effects of CM for drug use during opiate addiction treatment were observed to be moderated by five factors (type of reinforcer, time to reinforcement delivery, targeted CM drug(s), number of urine specimens collected per week and type of subject assignment). However, this review did not search the literature systematically, increasing the risk of bias in the selection of study data. Similarly, it did not assess the effects of different drugs targeted with CM, instead only assessing the moderating effects of targeting single or poly drug use. The aim of the present review was to assess the efficacy of CM for treating the use of different non-prescribed drugs during treatment for opiate addiction, by systematically searching the literature and assessing the effects of potentially moderating variables.

## 2 Method

A protocol for the current review is available online (see appendix of Supplementary file).

### 2.1 Search strategy

The review was carried out in accordance with PRISMA (Preferred Reporting Items for Systematic Reviews and Meta-Analyses) guidelines (Moher, 2009). Studies were identified using a keyword search of the online databases Embase; PsychInfo; PsychArticles using the Ovid SP interface and a MeSH search of Medline using the PubMed interface; with the following search terms: “Contingency Management” **or** “Reward” **or** “Payment” **or** “Incentive” **or** Prize” **and** “Substance” **or** “Misuse” **or** “Drug” **or** “Narcotic*” **or** “Tobacco” **or** “Smok*” **or** “Stimulan*” **or** “Cocaine” **or** “Alcohol” **and** “Opiate” **or** “Opioid” **or** “Heroin” **or** “Methadone”. The search was limited to studies published between each database’s inception and March 2015; published in the English language and including only humans. See appendix^1^ for full search strategy.

### 2.2 Inclusion and exclusion criteria

Studies were eligible for inclusion if they: *i)* Tested one or more CM intervention(s) aimed at substance use reduction or abstinence in patients receiving treatment for opiate addiction. CM included any intervention that consistently administered rewards to positively reinforce substance use reduction or abstinence in patients receiving treatment for opiate addiction; *ii)* used a controlled trial design–either a no/delayed treatment control group or an alternative therapy control group, or controlled by repeated participation in two or more treatment arms; *iii)* randomised participants to conditions*; iv)* provided reinforcement or punishment contingent on biological verification of substance use/abstinence; *v)* used consistent measures of substance use at baseline and follow-up; *vi)* Published in a peer reviewed journal. Studies were excluded if: *i)* Participation was non-voluntary – e.g., court orders, prison inmates etc.; *ii*) means and standard deviations for treatment effects were not available from the published data or the authors.

### 2.3 Study selection

Studies were reviewed for inclusion by three independent reviewers, with all studies being reviewed for inclusion twice. TA processed all titles and abstracts as first reviewer, RC and LB jointly processed half each as second reviewers. An agreement rate of 96% was reached between reviewers; disagreements were discussed and resolved by a separate reviewer, AM.

### 2.4 Quality assessment

The ‘Quality Assessment Tool for Quantitative Studies’ (Effective Public Health Practice Project, 2003) was used to assess the internal and external validity of all studies, as well as any biases and confounds. This assesses the quality of studies as strong, moderate or weak on six domains (selection bias, study design, confounds, blinding, data collection and withdrawals/dropouts), providing an overall score for the quality of the evidence in the study. A study is rated as providing strong evidence only when all domains are rated as moderate or strong, and a moderate rating when strong or moderate ratings are achieved for all bar one of the domains. Inter-rater reliability has been shown to be ‘fair’ across the six domains and ‘excellent’ overall, often performing better than the Cochrane Collaboration Risk of Bias Tool (Armijo-Olivo et al., 2012).

### 2.5 Data extraction and synthesis

All data extraction was completed by a single reviewer (TA) using an extraction table designed specifically for the current review and agreed by all reviewers (see supplementary materials). Where studies did not contain means and standard deviations for treatment effects, authors were contacted up to two times to obtain the data. Requests for data were sent to authors of 35 studies, with data for six studies being received (Carpenedo et al., 2010, Downey et al., 2000, Epstein et al., 2009, Kirby et al., 2013, Petry et al., 2007, Vandrey et al., 2007). Where means and standard deviations were not obtained, alternative data including F tests, *t*-tests and chi square were used to calculate an effect size where feasible (Dunn et al., 2010, Shoptaw et al., 2002, Silverman et al., 1998, Silverman et al., 1996).

### 2.6 Outcome measures

Standardised mean differences (Cohen’s *d* (Cohen, 1988)) were calculated for each individual study using either: 1) longest duration of abstinence (LDA) data or 2) percentage of biochemically verified negative samples (PNS). As follow-up data were available for only three of the 10 studies that included a follow-up period, all data used in analyses are those recorded during treatment.

### 2.7 Moderators

A number of possible moderators were assessed, based on those shown in previous reviews to impact on the efficacy of CM (Griffith et al., 2000, Prendergast et al., 2006). These included the drug targeted for intervention, the decade in which the study was carried out, the quality of the study, duration of the intervention, the type of reinforcer used, and the form of opiate treatment participants were undergoing. Some moderators previously suggested to affect the efficacy of CM (Griffith et al., 2000, Prendergast et al., 2006) could not be investigated due to a lack of suitable data in the included studies or because all studies used the same approach. For example, the number of times abstinence was verified per week could not be investigated as 16 studies recorded this three times a week compared to only five recording it twice a week and one study recording it every day. Similarly, type of incentive (positive, negative, mixed) was not tested as all bar two studies in both analyses used a mixed incentive. Time to reinforcement could not be tested as all included studies delivered immediate reinforcements.

### 2.8 Data analysis

Meta-analyses were carried out using RevMan v5.3 (Cochrane Collaboration, 2014) software. Data were entered into a generic inverse variance analysis in RevMan that analysed the efficacy of CM compared with control across all drug use during treatment for opiate addiction, using both LDA and PNS. All meta-analyses were carried out as random effects analyses due to the wide variety of CM interventions included (Riley et al., 2011). To allow comparison of CM to control, some multi-arm trials were collapsed into a two-arm design by averaging the effects across the treatment conditions (Cochrane Colaboration, 2011). This was only done however when each arm used CM in isolation (other than normal pharmacological treatment for opiate addiction); if a study arm included CM in combination with another behavioural or pharmacological treatment not part of standard treatment, then this arm was not included in the meta-analysis. This was done in order to match the design of the included studies with only single experimental and control arms. Control arms were not collapsed unless each was a standard treatment control. For example, one study (Schottenfeld et al., 2005) had four conditions (CM with either methadone or buprenorphine and performance feedback with either methadone or buprenorphine), so the two CM conditions were collapsed together, as were the two performance feedback conditions. Another study (Preston et al., 2000) also had four conditions (CM, methadone increase, CM + methadone increase and a usual care control), but no conditions were collapsed and only the CM and usual care control conditions were used in the analysis. The *I^2^* statistic was used to assess the percentage of variability in treatment effect estimates attributable to between-study heterogeneity.

Moderator analysis was performed using Comprehensive Meta-analysis software V.3 (Borenstein et al., 2014). Results were computed using random effects statistics and indicate the extent to which each moderator accounts for variability in effect sizes with respect to drug use outcomes. A significant value of Q-between indicates significant differences among effect sizes between the categories of the moderator variable. This method also calculates the mean pooled effect size for each category within the moderator variable being tested and whether this is significant. For the drug targeted for intervention, studies fell into five categories: opiates, cocaine, opiates and cocaine combined, tobacco, and polysubstance use. For study decade, studies were grouped as being published from 1990 to 1999, 2000 to 2009 and 2010 onwards (study publication dates ranged from 1993 to 2015). Study quality followed the strong, moderate and weak ratings of the ‘Quality Assessment Tool for Quantitative Studies’ (Effective Public Health Practice Project, 2003). Intervention durations were grouped as <12 weeks, 12 weeks, and >12 weeks. Reinforcer type was categorised as monetary vouchers and ‘other’. Opiate treatment similarly contained two categories, methadone treatment and ‘other’.

Publication bias was assessed using the ‘failsafe N’ technique (Rosenthal, 1979), calculated using Comprehensive Meta-analysis software V.3 (Borenstein et al., 2014). This calculates the number of studies averaging a Z-value of zero that would be required to make the overall pooled effect size non-significant (Rosenthal, 1979).

## 3 Results

### 3.1 Included studies

**Fig. 1:**
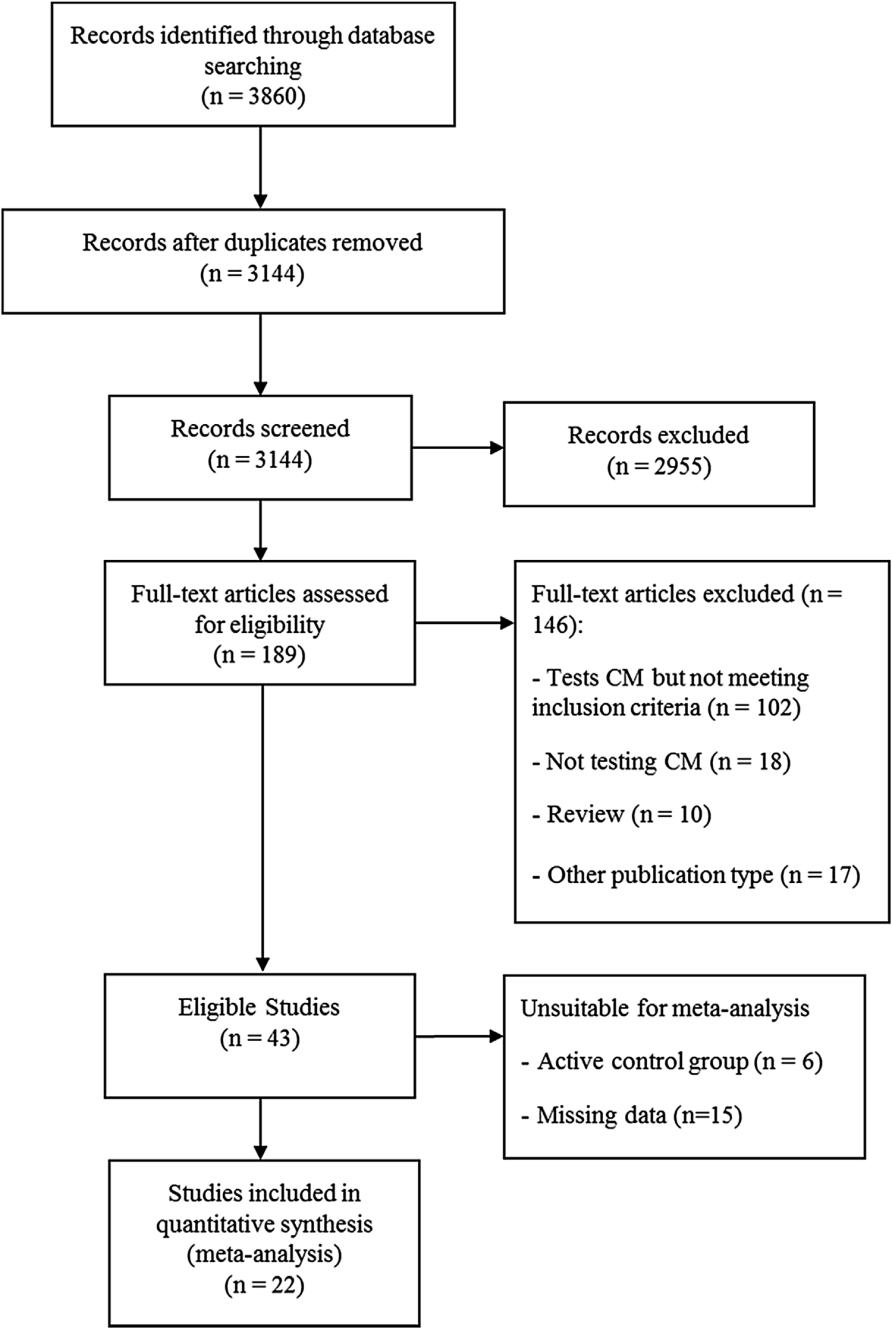
PRISMA flow diagram.

A total of 3144 studies were identified in the search, yielding a total of 22 studies meeting inclusion criteria and included in the meta-analysis (Chutuape et al., 2001, Chutuape et al., 1999, Downey et al., 2000, Dunn et al., 2010, Epstein et al., 2009, Epstein et al., 2003, Gross et al., 2006, Katz et al., 2002a, Katz et al., 2002b, Kidorf and Stitzer, 1993, Kidorf and Stitzer, 1996, Ling et al., 2013, Peirce et al., 2006, Petry et al., 2014, Petry et al., 2007, Petry and Martin, 2002, Preston et al., 2000, Schottenfeld et al., 2005, Silverman et al., 1998, Silverman et al., 1996, Umbricht et al., 2014, Vandrey et al., 2007) (see PRISMA flow diagram, Fig. 1). The included studies randomised a total of 2333 patients to 39 CM conditions and 33 non-CM control conditions. This included three studies with two CM conditions each collapsed into a single CM condition, four studies with three CM conditions each collapsed into a single CM condition, and two studies with two CM, and two control, conditions each collapsed into single CM and control conditions.

### 3.2 Study description and quality assessment

Eight of the 22 studies tested the effects of CM for cocaine use, two for opiate use, one for tobacco smoking, six for combined use of opiates and cocaine and five for polysubstance use. Twenty-one studies included some form of opiate substitution therapy (18 methadone, one buprenorphine, one a mixed buprenorphine and naloxone tablet, and one suboxone), with only a single study not utilising any form of opiate substitution therapy. The duration of CM interventions used ranged between 11 days and 31 weeks, with the number of participants in each study ranging between 12 and 388. Seventeen studies reported retention rates, resulting in an average retention rate of 76.4% (range 51.2%–97.7%). All studies were carried out in the US, with 13 being carried out in the same state (Maryland) (See Table 1 for full description of studies and interventions). Methodological quality assessment rated two studies as overall providing strong evidence, 10 studies moderate evidence and 10 studies weak evidence (Table 2).

**Table 1:** Description of each included study and intervention, organised by drug target of CM intervention.

Study, publication date, publishing journal and location carried out	Design and usual opiate substitution therapy treatment	Participants randomised pre and post intervention	Intervention procedure	CM Schedule, length of intervention and max reward	Additional treatments	Primary Outcome	Abstinence Criteria	Substance use post intervention	Substance use at longest follow up
Cocaine									
Epstein et al. (2003)	2 × 2 factorial design. CM or no CM, and CBT or Social support	Rand − 193	Urines collected every Mon, Wed and Fri, and vouchers administered dependent on condition	Escalating with reset and bonus for three consecutive negative samples	Individual counselling sessions focussing on cessation of all drugs	Number of drug negative urines	Benzo <300 ng/ml	Throughout intervention, BZE levels were lower in the CM-only and combination groups than in the other two groups. F(1, 185) = 15.94, p < 0.001	No significant difference between any of the groups at 12 month follow up
Psychology of Addictive Behavior Baltimore, Maryland, USA	Meth., between 50 and 80 mg/day	Post − 147		12 Weeks					
				Max $1155					
Katz et al., 2002a, Katz et al., 2002b		Rand − 40		Multiple Each phase lasted 11 days			50% reduction in Benzo. or Benzo <300 ng/ml		
Experimental and Clinical Psychopharmacology Baltimore, Maryland, USA	Repeated measures − single, continuous, interrupted or no voucher meth. 100 mg/day	Post − Not reported	Urines collected Mon, Wed and Fri. Vouchers awarded dependent on condition (one large voucher, continuous or interrupted vouchers, or no voucher)	Max reward dependent on condition	Weekly individual and group counselling	Number of consecutive days cocaine abstinence	LDA	Mean abstinence duration was 2 days for no voucher, 3.2 days for single-voucher, and 4.9 and 4.8 days for continuous and interrupted voucher conditions, respectively, F(3, 117) = 7.3, p = < 0.001.	N/A
Kidorf et al. (1993)		Rand − 44		Fixed schedule			Definition not reported		
				7 Weeks					
Experimental and Clinical, Psychopharmacology Baltimore, Maryland, USA	CM or Yoked Control group. Ppt were accepted into the 2 years meth. treatment once the exp had done so Meth. 50 mg/day	Post − 43	Urines collected Mon, Wed and Fri. The single reward was awarded after two consecutive weeks of cocaine abstinence which had to occur within the 7 week probationary period	Single reward of 2 years meth. treatment	Group and individual counselling at least once per week	Two consecutive weeks of cocaine abstinence	PNS	50% of CM and 14% of control achieved 2 weeks of continuous cocaine abstinence. No significant difference was found between conditions for the number of negative urines returned	No significant difference between the two conditions was found for the proportion of cocaine negative urines submitted
Petry et al. (2007)		Rand − 76		Fishbowl or voucher escalating with reset.			Not reported		
Journal of Consulting and Clinical Psychology, Connecticut, USA	Prize based (fishbowl) or voucher based CM, or standard care control Meth. Mean dose between 78.4 and 83 mg/day dependent on condition	Post − 59	Urines collected twice per week with an average of 4 days between submissions. Negative samples resulted in draws from the prize earn, or vouchers.	12 weeks Max up to $300 and $585 respectively	Weekly individual and/or group counselling	Cocaine abstinence	LDA and PNS	Fishbowl CM ppt achieved significantly greater LDA than control ppt. Voucher CM ppt did not.	No significant difference between percentage of participants submitting negative samples in any condition at 9 months
Silverman et al. (1998)		Rand −59		Escalating with reset, with bonuses in one condition. 12 weeks			Benzo. <300 ng/ml		
Journal of Consulting and Clinical Psychology Baltimore, Maryland, USA	Three conditions, Escalating CM, Escalating CM with start bonus, and yoked control Meth. Mean dose 62 mg/day	Post − Average retention 10.3–11.3 weeks dependent on condition	Urines collected Mon, Wed and Fri. Vouchers dispensed after urines tested	Max reward $1950 without bonuses	Offered weekly individual counselling	Not reported	LDA	Both CM conditions achieved significantly longer durations of abstinence	Difference between CM groups and control remained significant at 8 weeks
Silverman et al. (1996)	Two conditions, escalating with reset CM and yoked control	Rand − 37	Urines taken Mon, Wed and Fri.	Escalating with reset and bonus.			Benzo. <300 ng/ml		
				12 weeks					
Archives of General Psychiatry, Baltimore, Maryland, USA	Meth. 50 mg/day	Post − 89% of exp ppt and 83% of ctrl ppt retained for full 12 weeks	Vouchers given for abstinence	Max $1155	Weekly individual counselling (45 min per week)	Not reported	LDA	Exp patients achieved significantly longer durations of sustained cocaine abstinence than ctrl ppt (F(1.35) = 13.5; p = <0.001)	No significant difference found between groups 4 weeks post intervention
Umbricht et al. (2014)	2 × 2 Design. CM or Yoked control and Topiramate or placebo.	Rand − 171		Escalating with reset.			Benzo. <300 ng/ml		
Drug and Alcohol Dependence, Baltimore, Maryland, USA	Meth. 100 mg/day	Post − 113	Urines collected Mon, Wed and Fri. Vouchers awarded for abstinence	31 weeks Max $1155	Weekly individual and group counselling	Cocaine abstinence between weeks 9 and 20	PNS and LDA	No significant difference found between any of the conditions	N/A
Vandrey et al. (2007)		Rand − 12		Fixed, with a single voucher or cheque available in each condition.			Benzo. <300 ng/ml		
Experimental and Clinical Psychopharmacology	2 × 4 design − 2 types of reward type (voucher or cheque) and 4 types of reward magnitude ($0, $25, $50 or $100) Meth., dose not reported	Post − Not reported	Urines collected Mon, Wed and Fri. Rewards were provided for evidence of abstinence Mon to Wed, on the Thur	16 weeks (two 8 week periods) Largest voucher value $100	Group and individual counselling	Not reported	PNS	No main effect of incentive type. Planned comparisons found that high value cheques resulted in significantly greater abstinence than high value vouchers	N/A

Opiates									
Ling et al. (2013)	4 conditions, 4 CM, CBT, CM + CBT and no behavioural treatment Control	Rand − 202		Fishbowl with escalating draws.			Exact criteria not reported		
Addiction, Los Angeles, USA	Suboxone, variable dose	Post − 134	Urines collected twice weekly, with escalating numbers of draws for vouchers dependent on drug free urines	16 weeks Max initially $2196, later reduced to $14600	Counselling	Proportion of opiate negative urines	PNS	Mean number of consecutive opioid-negative UA results did not differ significantly by group.	Same results 52 week follow up as post treatment
Preston et al. (2000)		Rand − 120		Escalating with reset.			<300 ng/ml opiates		
				8 weeks					
Archives of General Psychiatry, Baltimore, Maryland, USA	4 Conditions: CM, Increased meth. with non contingent vouchers, CM + meth. increase, usual treatment control with non contingent vouchers Meth. dose not reported	Post − 112	Urines collected Mon, Wed and Fri. Vouchers administered for evidence of abstinence	Max $554	Weekly individual counselling	Opiate negative urine samples	PNS and LDA	LDA significantly increased with contingent vouchers (F(1116) = 10.02, p = 0.002)	N/A

Cocaine and Opiates									
Chutuape et al. (2000)		Rand − 53		Escalating with reset.					
Drug and Alcohol Dependence, Baltimore, Maryland, USA	3 conditions: CM with weekly or monthly urine testing, and a control where take home meth. was awarded randomly Meth. 60 mg/day	Post − 43	Urines collected Mon, Wed and Fri. One urine randomly selected either weekly or monthly dependent on condition to decide whether vouchers awarded	28 weeks Max reward was take home doses for all weeks	Weekly individual and group counselling sessions	Not reported	Not reported	The mean LDA was 10.5 (SD 8.9), 8.4 (SD 8.5), and 5.4 (SD 7) weeks for the Weekly, Monthly, and Random Drawings groups, respectively (F(2.52) 1.9, PB0.16).	N/A
Epstein et al. (2009)		Rand − 252		Escalating with reset.			<300 ng/ml for both opiates and cocaine		
Drug Alcohol Dependence, Baltimore, Maryland, USA	3 × 2 dose by contingency design − meth. dose of either 70 mg or 100 mg and yoked control, CM for cocaine or split CM for cocaine and opiates	Post − 23% of ppt dropped out before the end of the intervention	Urines collected Mon, Wed and Fri. Vouchers were awarded for abstinence from cocaine and opiates either together or separately dependent on condition	12 weeks Max not reported	Weekly individual counselling	Percentages of urine specimens negative for heroin, cocaine, and both simultaneously	PNS and LDA	Main effect of contingency on cocaine-negative urines, (F(2244) = 7.36, p = 0.0008) and on urines simultaneously negative for opiates and cocaine, (F(2244) = 3.61, p = 0.0285) but not in opiate-negative urines, (F(2244) = 2.51, p = 0.0830)	N/A
Groß et al. (2006)	Three conditions: CM vouchers, Reduction in medication, and standard treatment control	Rand − 60		Escalating with reset and bonus.			<300 ng/ml of cocaine or opiates		
Experimental and Clinical Psychopharmacology, Vermont, USA	Bup, maintained on either 4 mg/70 kg or 8 mg/70 kg for the duration of the study	Post − 45	Urines collected Mon, Wed and Fri. Dependent on condition, ppt either earned points, or did not have their bup dose decreased on evidence of abstinence	12 weeks Max $269	Behavioural drug counselling	Mean duration of continuous abstinence, total number of weeks abstinent (non-continuous), and number of missing visits.	LDA	Contingent medication ppt achieved significantly greater durations of continuous abstinence (M = 5.9 weeks, SD = 4.6) than ppt in the voucher group (M = 2.9 weeks, SD = 3.3; Fisher’s LSD, p=0.05).	N/A
Katz et al. (2002a,b)	Two conditions, CM or Standard care	Rand − 52					<300 ng/ml for both opiates and cocaine		
Experimental and Clinical Psychopharmacology, Baltimore, Maryland, USA	Meth. 100 mg/day	Post − Mean 35.9 days (of 180) in treatment	Urines collected three times per week and vouchers administered for negative samples	Escalating with reset and bonus 12 weeks Max $1,087.50	Weekly individual cognitive behavioural counselling	Not reported	LDA and PNS	No statistically significant condition effects found	N/A
Petry et al. (2002)	CM or standared treatment	Rand − 42		Fishbowl, escalating draws.			Not reported		
Journal of Consulting and Clinical Psychology, Connecticut, USA	Meth. Average 69 or 70 mg/day in standard treatment and CM	Post − 39	Urines collected Mon, Wed and Fri. Ppt received on draw for abstinence from either cocaine or opiates, and four for abstinence from both. Continuous weekly abstinence earned bonus draws	12 weeks Max number of draws dependent on abstinence from different drugs	Monthly individual counselling	Weeks of continuous abstinence from both opioids and cocaine	LDA	There were significant group difference in the percentage of urine samples negative for both drugs (F(1, 40) = 4.01, p = 0.05	The percentage of urine samples negative for both opioids and cocaine was higher in exp than ctrl ppt (U = 112.0, p=0.05.) at 6 month follow up
Schottenfeld et al. (2005)		Rand − 162		Escalating with reset.			<300 ng/ml for both opiates and cocaine		
The American Journal of Psychiatry, USA	2 × 2 design: meth. or buprenorphine and CM or performance feedback Maximum daily meth. dose of 85 mg or bup. dose of 16 mg	Post − Cumulative proportion: meth. + CM − 0.6, meth. + performance feedback − 0.75, Bup + CM − 0.45, Bup + Performance feedback − 0.5	Urines collected Mon, Wed and Fri and vouchers administered for evidence of abstinence	24 week Max $1033.50	Individual counselling twice weekly for the first 12 weeks and weekly for the last 12	Maximum number consecutive weeks of abstinence and proportion of drug-free urine tests	LDA	meth. ppt achieved significantly longer periods of abstinence than bup. There were no significant effects of CM (F = 0.09, df = 1, 158, p = 0.76) and no significant interaction between medication and CM (F = 0.10, df = 1, 158, p = 0.75)	N/A

Tobacco									
Dunn et al. (2010)		Rand − 40		Escalating with reset 90 days					
Experimental and Clinical Psychopharmacology Vermont, USA	Two conditions: CM and non contingent voucher Meth. 107.6 ± 8.8 mg/day or Bup. 14.9 ± 1.3 mg/day	Post − 25	Biochemical verification taken everyday with vouchers for abstinence delivered daily. Numerous bonus's available for abstinence at certain points	Max $362.50	None reported	Percentage of biochemical samples meeting abstinence criteria	Abstinence defined as breath CO ≤ 6 ppm during days 1–5 and a urine cotinine ≤ 80 ng/ml on Days 6–14 PNS and LDA	Exp. Ppt submitted significantly more negative samples than ctrl. Ppt (t (30.1) = 3.24, p < 0.01)	No significant difference between the two conditions at any follow up

Poly substance use									
Chutuape et al. (1999)	Two conditions: CM and usual care control	Rand − 14					<200 ng/ml for meth., opiates, cocaine and benzodiazepines		
Drug and Alcohol Dependence, Baltimore, Maryland, USA	Meth. 71 mg/day or 77 mg/day in CM and standard care conditions	Post − 12	Urines collected Mon, Wed and Fri. Vouchers or take homes administered for evidence of abstinence dependent on ppt choice	Fixed. 12 weeks Max $900 or three take homes per week dependent on ppt choice	Twice-weekly counselling sessions (one individual and one group session)	Number of drug free urines	LDA	mean LDA for exp ppt was 8.4 and 1 week for ctrl ppt (t(8) = 5.9, p = <0.001.)	5 ppt relapsed after the CM intervention. ended, generally within the first week
Downey et al. (2000)	Two conditions: CM and Yoked control	Rand − 41	Urines taken Mon, Wed and Fri.	Escalating with reset and bonus.			<300 ng/ml for all drugs other than phencyclidine which was <25 ng/ml		
Experimental and Clinical Psychopharmacology, USA	Mixed Bup. Naloxone tablets. Dose not reported	Post − 21	Vouchers administered for evidence of abstinence	12 weeks Max not reported	Weekly cognitive behavioural substance abuse therapy	Not reported	LDA	No sig difference between the two groups on% drug free urines, LDA or total abstinence for heroin, cocaine or poly drug use during the voucher phase	N/A
Kidorf et al. (1996)		Rand − 16		Fixed with negative consequences for drug positive samples.					
Behavior Therapy, Baltimore, Maryland, USA	Two conditions: CM and usual care control Meth. 60 mg/day	Post − 14	Urines collected Twice per week and take homes administered for evidence of abstinence. Samples positive for drugs resulted in meth. being administered in a split dose	2 month cross over Max 2 take homes per week	Weekly individual counselling	Percentage of drug free urines	Breath alcohol < 0.5, other drug cut-offs not reported PNS	A condition main effect was found, (F(2, 30) = 4.43, p = < 0.05.) Patients submitted more drug-free urines when exposed to exp (M = 29%; SE = 9.0) than ctrl (M = 9%; SE = 3.0)	N/A
Peirce et al. (2006)		Rand − 388		Fishbowl, escalating with reset.			Not reported		
Archives of General Psychiatry USA	Two conditions: CM and usual care control Meth. Doeses ranging between 67.9 mg/day to 108 mg/day dependent on recruitment centre	Post − 67.1% of exp ppt and 64.8% ctrl ppt retained	Urines collected twice per week and prize draws allowed for evidence of abstinence	12 weeks Max 204 draws, resulting in a maximum of approx. $400 in prizes, plus one guaranteed $20 prize.	Individual and group consoling. Frequency ranged from 3 times per week to once per month	Not reported	LDA	Exp ppt were significantly more likely to submit stimulant- and alcohol-negative samples than were ctrl ppt (OR, 1.98; 95% CI, 1.42-2.77; missing samples coded as missing)	No group differences in percentage of submitted samples negative for stimulants and alcohol (χ^2^ = 0.08, P=0.78).
Petry et al. (2015)		Rand − 240		Escalating with reset for either fishbowl draws or vouchers dependent on condition.			Not reported		
Journal of Consulting and Clinical Psychology, USA	Four conditions: $300 prize CM, $900 prize CM, $900 voucher CM and usual care control Meth. Doses ranging between 77 mg/day and 85.4 mg/day	Post − Not reported	Urines taken at least twice a week with at least 2 days between tests. Abstinence resulted in either fishbowl draws or vouchers	12 weeks Max either $300 or 900$	Weekly group counselling	LDA and proportion of samples submitted negative for cocaine and alcohol	PNS and LDA	The longest duration of abstinence and proportion of samples testing negative were significantly greater in each of the three CM conditions relative to usual care (F(3236) = 3.39, p = 0.02 and F(3236) = 3.94, p=0.009 respectively)	At the 12-month follow-up, 113 of 225 (50.2%) patients submitted negative samples

Abbreviations – **Rand**- Randomised to conditions, **Post**- Post intervention, **Exp** – Experimental condition(s), **Ctrl** – Control condition, **CM** – Contingency Management, **TLFB** – Time Line Follow Back, **LDA** – longest duration of abstinence, **PNS** – percentage of negative samples, **Meth.** – methadone, **Bup.** – buprenorphine, **Pbo.** – placebo, **ppt** – participants, **Benzo** – benzoylecgonine, **OST** – Opiate substitution therapy.

**Table 2:** EPHPP ratings for all included studies organised by drug target of CM intervention.

Study	Selection Bias	Study Design	Confounds	Blinding	Data Collection	Withdrawals/ Dropouts	Overall
Cocaine							
Epstein et al. (2003)	2	1	1	2	1	2	Strong
Katz et al., 2002a, Katz et al., 2002b	2	1	3	2	1	1	Moderate
Kidorf et al. (1993)	3	1	1	2	1	1	Moderate
Petry et al. (2007)	3	1	1	3	1	2	Weak
Silverman et al. (1996)	3	1	1	2	1	1	Moderate
Silverman et al. (1998)	2	1	1	2	1	3	Moderate
Umbricht et al. (2014)	3	1	1	1	1	2	Moderate
Vandrey et al. (2007)	3	1	3	2	1	3	Weak

Opiates							
Ling et al. (2013)	2	1	3	2	1	2	Moderate
Preston et al. (2000)	3	1	3	1	1	1	Weak

Opiates and Cocaine							
Chutuape et al. (2000)	3	1	1	2	1	3	Weak
Epstein et al. (2009)	3	1	1	2	1	2	Moderate
Groß et al. (2006)	3	1	1	2	1	2	Moderate
Katz et al., 2002a, Katz et al., 2002b	2	1	1	2	1	3	Moderate
Petry et al. (2002)	2	1	1	2	1	1	Strong
Schottenfeld et al. (2005)	3	1	1	1	1	3	Weak

Tobacco							
Dunn et al. (2010)	2	1	1	3	1	2	Moderate

Poly-substance							
Chutuape et al. (1999)	3	1	3	2	1	3	Weak
Downey et al. (2000)	3	3	3	2	1	3	Weak
Kidorf et al. (1996)	3	1	3	2	1	3	Weak
Peirce et al. (2006)	3	1	1	3	1	2	Weak
Petry et al. (2015)	3	1	1	2	1	3	Weak

1 = Strong, 2 = Moderate, 3 = Weak

### 3.3 Meta-Analysis

**Fig. 2:**
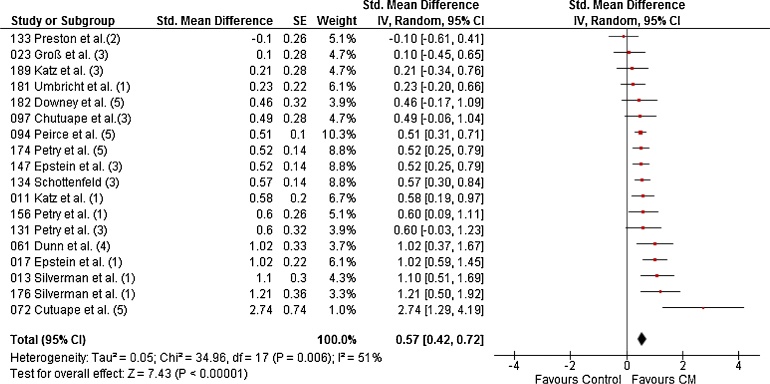
Forest plot for LDA during treatment of all substances combined. (1) = Cocaine, (2) = opiates, (3) = opiates and cocaine, (4) = Tobacco, (5) = Poly-substance.

The meta-analysis for LDA (longest duration of abstinence) from all substances combined contained 18 studies randomising 2059 patients to 31 CM conditions and 25 non-CM control conditions. The random effects meta-analysis produced a pooled effect size of *d* = 0.57 (95% CI: 0.42–0.72), with CM performing significantly better than control (Fig. 2). A moderate (Cochrane Colaboration, 2011) level of the variability of effects between studies was due to between-study heterogeneity (I^2^ = 51%).

**Fig. 3:**
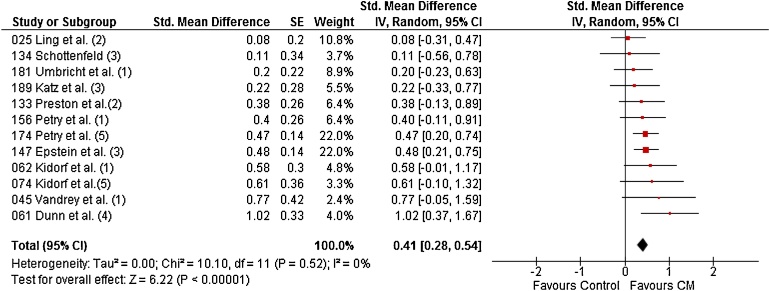
Forest plot for PNS during treatment of all substances combined. (1) = Cocaine, (2) = opiates, (3) = opiates and cocaine, (4) = Tobacco, (5) = Poly-substance.

For PNS (percentage of negative samples), 12 studies randomising 1387 patients to 24 CM conditions and 21 non-CM control conditions were included and the pooled effect size was *d* = 0.41 (95% CI: 0.28–0.54), again with CM performing significantly better than control (Fig. 3). Variability of effects was not due to between-study heterogeneity (I^2^ = 0%).

### 3.4 Moderator analysis

The only moderator found to have a significant effect on the efficacy of CM was intervention drug target, but only for LDA (Table 3, Table 4). Within each of the categories of the six moderators, CM performed significantly better than control in all but three instances. Within drug targeted for intervention, CM performed no better than control for treating non-prescribed opiate use for both LDA and PNS. Within intervention duration, CM failed to encourage significantly better LDA than control in studies with intervention duration of less than 12 weeks. Within opiate treatment type, CM did not result in significantly greater PNS than control for studies where participants were in the ‘other’ category.

**Table 3:** Random effects moderator analysis results for LDA.

Moderator	k^1^	Effect Size (d)^2^	95% CI	Z Value	P value	Q between (df)^3^	P of Q between
Drug targeted for intervention	18					10.75 (4)	0.03
Cocaine	6	0.75	0.45–1.04	4.91	<0.001		
Opiates	1	−0.10	−0.61–0.41	−0.40	0.70		
Opiates and cocaine	6	0.48	0.32–0.64	5.85	<0.001		
Tobacco	1	1.02	0.37–1.67	3.10	<0.01		
Poly substance	4	0.62	0.27–0.98	3.45	<0.01		

Study decade						1.31 (2)	0.52
1990–1999	4	1.08	0.14–2.02	2.23	0.02		
2000–2009	10	0.53	0.41–0.65	8.67	<0.001		
2010 onwards	4	0.53	0.32–0.74	4.92	<0.001		

Study Quality						2.66 (2)	0.23
Stong	2	0.87	0.48–1.27	4.37	<0.001		
Moderate	8	0.57	0.32–.82	4.47	<0.01		
Weak	8	0.51	0.30–0.72	4.75	<0.001		

Intervention Duration						1.30 (2)	0.52
<12 Weeks	2	0.26	−0.41–0.93	0.77	0.44		
12 Weeks	12	0.63	0.44–0.82	6.42	<0.001		
>12 Weeks	4	0.53	0.27–0.79	4.04	<0.001		

Reinforcer type						0.022	0.88
Monetary Vouchers	16	0.57	0.41–0.74	6.86	<0.001		
Other'	2	0.54	0.13–0.95	2.55	0.01		

Opiate treatment						0.65	0.42
Methadone	13	0.61	0.42–0.80	6.45	<0.001		
Other	5	0.47	0.20–0.74	3.46	<0.01		

^1^Number of studies, ^2^Weighted random effects, ^3^A significant value of Q-between indicates significant differences among effect sizes between the categories of the moderator variable

**Table 4:** Random effects moderator analysis results for PNS.

Moderator	k^1^	Effect Size (d)^2^	95% CI	Z Value	P value	Q betweeen (df)^3^	P of Q between
Drug targeted for intervention						6.43 (4)	0.17
Cocaine	4	0.4	0.13–0.67	2.89	<0.01		
Opiates	3	0.18	−0.11–0.46	1.23	0.22		
Opiates and cocaine	2	0.43	0.18–0.67	3.42	<0.01		
Tobacco	2	1.02	0.37–1.67	3.09	<0.01		
Poly substance	1	0.49	0.23–0.74	3.74	<0.001		

Study decade						1.10 (2)	0.58
1990–1999	2	0.51	0.25–0.77	3.83	<0.001		
2000–2009	3	0.30	0.01–0.59	2.01	0.05		
2010 onwards	7	0.40	0.20–0.60	3.93	<0.001		

Study Quality						0.36 (2)	0.84
Stong	1	0.48	0.21–0.75	3.43	<0.01		
Moderate	5	0.36	0.06–0.66	2.32	0.02		
Weak	6	0.44	0.30–0.58	0	<0.001		

Itntervention Duration						0.32 (2)	0.85
<12 Weeks	5	0.47	0.28–0.67	4.73	<0.001		
12 Weeks	2	0.42	0.18–0.67	3.35	0.04		
>12 Weeks	5	0.37	0.02–0.71	2.06	<0.01		

Reinforcer type						0.41 (1)	0.52
Monetary Vouchers	9	0.39	0.23–0.54	4.82	<0.001		
Other'	3	0.51	0.17–0.85	2.94	<0.01		

Opiate treatment						0.35 (1)	0.55
Methadone	8	0.45	0.30–0.60	6.00	<0.001		
Other	4	0.32	−0.08–0.72	1.58	0.12		

^1^Number of studies, ^2^Weighted random effects, ^3^A significant value of Q-between indicates significant differences among effect sizes between the categories of the moderator variable.

### 3.5 Publication bias

There is widespread acceptance of the fact that studies reporting positive results are far more likely to be published than studies reporting null findings, resulting in an over representation of positive results within the literature (Rosenthal, 1991, Rosenthal and Rubin, 1988, Schmid, 2016). The ‘failsafe N’ (Rosenthal, 1979) calculates the number of studies reporting null results that would be required to overturn the statistically significant difference between CM and control observed above. For LDA, 560 papers reporting null results would be required, and 101 for PNS.

## 4 Discussion

Overall, the random effects analyses showed CM performed significantly better than control in encouraging abstinence from a range of different drugs in patients undergoing treatment for opiate addiction. This was the case when measuring both LDA and PNS, producing medium and small (Cohen, 1988) pooled effect sizes respectively. Moderator analysis performed on drug targeted for intervention, decade in which the study was carried out, quality of the study, duration of the intervention, type of reinforcer used, and form of opiate treatment, showed drug target for LDA data to be the only characteristic significantly moderating the efficacy of CM, driven primarily by the ineffectiveness of CM in treating opiate use. Despite only a single significant moderator effect, within each of the six moderator categories CM was found to perform significantly better than control in all but three cases. CM performed no better than control in encouraging abstinence from non-prescribed opiates during treatment for opiate addiction, measuring both LDA and PNS. CM also performed no better than control for LDA in studies with interventions less than 12 weeks long, and PNS in studies where usual opiate treatment was anything but methadone treatment. CM for other non-prescribed drug use in treatment for opiate addiction had no negative impact on usual treatment retention compared to three-month follow-up retention rates observed in usual opiate treatment (Burns et al., 2015, Hansen et al., 1990, Soyka et al., 2008).

This review has a number of limitations. One aim of the moderator analysis was to analyse the effects of CM by target drug type. To improve on the work of Griffith et al. (2000), five categories of drugs were used rather than two. However, one of them, polysubstance use, combined studies with four differing definitions of this, making results hard to integrate. CM still performed better in this category though, suggesting a robustness of effects across a variety of different drug combinations. Another limitation is that the review does not contain any grey literature. This means that any CM studies that have been conducted yet never published are not included in the analysis.

The current review does have a number of strengths however. It is the first review in over 16 years to address directly the efficacy of CM for encouraging abstinence from non-prescribed drug use during treatment for opiate addiction. This is important as CM has gained considerable support in this time, having been recommended since 2007 as a treatment for drug misuse by the National Institute for Health and Care Excellence (Pilling et al., 2007). The findings of the current review support those of the previous reviews carried out in the field; finding an overall positive small to medium (Cohen, 1988) effect size for CM in treating drug use in opiate addiction treatment (Griffith et al., 2000). This is in contrast to the usual small effect size of psychological interventions in the field (Dutra et al., 2008). Findings of the present review are also similar to those of a previous reviews assessing the use of CM for drug use overall, regardless of treatment setting which found similar small to medium effect sizes for drug use in general (Benishek et al., 2014, Castells et al., 2009, Davis et al., 2016, Lussier et al., 2006, Prendergast et al., 2006). The robustness of the effects of CM across different client groups suggests potential utility in treating a diverse range of individuals and needs within the addictions field.

We found no evidence of CM working better than control in encouraging abstinence from non-prescribed opiates during treatment, which is in contrast to Prendergast et al. (2006) who identified CM as one of the most effective treatments for opiate use. The current review included only two studies of this type, compared to four (different) studies included in the previous review because of differing review aims. Moreover, three of the four opiate studies in the previous review systematically reduced methadone doses to zero over the course of the intervention, thereby increasing the likelihood of relapse to opiates and perhaps handing those receiving CM a competitive advantage over those not. Studies in the current review however maintained medication doses throughout the duration of the intervention, possibly eliminating this advantage and leading to the observed non-significant finding. With more data however, results for opiates may more closely follow the trends observed with other drugs.

The moderator analysis performed in the current review has also produced contradictory results to previous reviews. Previous reviews (Griffith et al., 2000, Prendergast et al., 2006) found four of the six moderators analysed here to have a significant effect on the efficacy of CM (drug targeted for intervention, the decade in which the study was carried out, the quality of the study evidence, the length of the intervention period). The current study only found a significant effect for drug targeted for intervention however. A possible explanation for this is differences in analysis, with the previous reviews adopting a fixed effects analysis, and the current the more conservative and more widely recommended (Cochrane Colaboration, 2011) random effects analysis. Support for this comes from more recent reviews that have adopted this same random effects analysis. Lussier et al. (2006) for example analysed the effects of three (drug targeted for intervention, the decade in which the study was carried out, the quality of the study evidence) moderators also analysed in the current and previous reviews, finding none of them to have a significant effect.

More general limitations within the field have also been identified, for example a lack of data available for meta-analysis. In the current review, a total of 21 studies that met all other inclusion criteria could not be included in the quantitative data synthesis. This lack of available data is even more pronounced for follow-up, with only 10 of the 22 included studies utilising some sort of follow-up element in their study design, with data available for only three. CM is often criticised for poor follow-up results, but given the paucity of data we were not able to explore this here. Another concern is the quality of the studies included, with only two studies being rated as providing strong evidence, and 20 papers providing weak evidence. Notably, every study in the current review was performed in the US, with at least 13 performed in the same state and 17 having at least one co-author from the same institution. This significantly limits the generalisability of the currently available evidence on CM for non-prescribed drug use in opiate addiction treatment.

This lack of evidence does however present avenues for future research, particularly the use of CM for tobacco smoking in opiate addiction treatment. This is especially relevant considering that tobacco smoking is the most prevalent form of drug use in opiate addiction treatment (Best et al., 2009, Clemmey et al., 1997), and it has been shown that individuals in treatment for opiate addiction treatment have a mortality rate four times that of non-smokers (Hser et al., 1994). It is similarly important that future research studies are carried out in a wider range of countries, include follow-ups to investigate relapse after the removal of rewards, and focus on improving the overall quality of the data that are published.

In conclusion, CM appears to be an efficacious treatment of the use of cocaine, non-prescribed opiates and cocaine, tobacco, and polysubstance use during opiate addiction treatment, but not for use of non-prescribed opiates. Evidence about longer-term efficacy in this treatment context remains lacking, as is research into the effects of CM on tobacco, the most prevalent secondary addiction in this population.

## Contributors

LB and RC acted as second reviewers during study selection. AM, LB and JS had editorial input during manuscript preparation. All authors approved of the final manuscript before submission.

## Role of funding source

This work was funded as part of TA’s PhD studentship by the Medical Research Council and the Institute of Psychiatry, Psychology and Neuroscience (MRC/IoP Excellence Studentship). Funding: LB is funded by a Cancer Research UK/BUPA Foundation Fellowship (C52999/A19748). All authors are part of the UK Centre for Tobacco and Alcohol Studies, a UK Clinical Research Collaboration Public Health Research: Centre of Excellence. Funding from the Medical Research Council, British Heart Foundation, Cancer Research UK, Economic and Social Research Council and the National Institute for Health Research under the auspices of the UK Clinical Research Collaboration is gratefully acknowledged (MR/K023195/1). The funders played no role in the study design, collection, analysis and interpretation of the data, in the writing of the manuscript and in the decision to submit this manuscript for publication. JS has previously received funding from the NIHR to test the application of contingency management in opiate addiction treatment.

## Conflict of interest

None declared.
